# Antiviral protection by antibodies targeting the glycan cap of Ebola virus glycoprotein requires activation of the complement system

**DOI:** 10.21203/rs.3.rs-2765936/v1

**Published:** 2023-04-21

**Authors:** Alexander Bukreyev, Philipp Ilinykh, Kai Huang, Bronwyn Gunn, Natalia Kuzmina, Pavlo Gilchuk, Galit Alter, James Crowe

**Affiliations:** University of Texas Medical Branch; University of Texas Medical Branch at Galveston; UTMB Galveston; Washington State University; University of Texas Medical Branch; Vanderbilt University Medical Center; The Ragon Institute of MGH, MIT and Harvard; Vanderbilt University Medical Center

## Abstract

Antibodies to Ebola virus glycoprotein (EBOV GP) represent an important correlate of the vaccine efficiency and infection survival. Both neutralization and some of the Fc-mediated effects are known to contribute the protection conferred by antibodies of various epitope specificities. At the same time, the role of the complement system in antibody-mediated protection remains unclear. In this study, we compared complement activation by two groups of representative monoclonal antibodies (mAbs) interacting with the glycan cap (GC) or the membrane-proximal external region (MPER) of the viral sole glycoprotein GP. Binding of GC-specific mAbs to GP induced complement-dependent cytotoxicity (CDC) in the GP-expressing cell line via C3 deposition on GP in contrast to MPER-specific mAbs that did not. Moreover, treatment of cells with a glycosylation inhibitor increased the CDC activity, suggesting that N-linked glycans downregulate CDC. In the mouse model of EBOV infection, depletion of the complement system by cobra venom factor led to an impairment of protection exerted by GC-specific but not MPER-specific mAbs. Our data suggest that activation of the complement system is an essential component of antiviral protection by antibodies targeting GC of EBOV GP.

## Introduction

Filoviruses include one of the deadliest human pathogens known to date. *Ebolavirus* genus of the *Filoviridae* family includes Ebola virus (EBOV), Sudan virus (SUDV), Bundibugyo virus (BDBV), Taï Forest virus (TAFV), Reston (RESTV) and Bombali virus (BOMV) [1]. EBOV, SUDV and BDBV are known to cause outbreaks and epidemics of highly lethal disease, which is often accompanied by hemorrhagic manifestations and systemic multiorgan dysfunction, with unpredictable periodicity, location, and scale [2]. The largest known ebolavirus epidemic took place in 2013–2016 in West Africa and was caused by EBOV. It claimed the lives of 11,310 out of 28,616 people infected [3].

Currently, monoclonal antibody (mAb) therapy has been shown to be the most effective treatment of filoviral infections after the onset of symptoms [4]. In 2020, two mAb-based therapeutics were developed and approved by the Food and Drug Administration for clinical use [5, 6] Notably, however, these therapeutics are only effective against EBOV but not other ebolaviruses. Therefore, efficacious treatments against other pathogenic filovirus species are urgently needed.

Ebolavirus glycoprotein (GP) is the sole envelope viral protein responsible for cell entry and, hence, serves as the primary target for antibody-based therapies and vaccine design efforts. EBOV GP precursor is a 676-residue, type I transmembrane protein. It is cleaved by the host subtilisin-like proprotein convertase furin in the Golgi into two subunits, GP1 and GP2, which remain associated through a disulfide bond [7]. The GP1/GP2 heterodimer assembles into a 450 KDa trimer at the surface of nascent virions. The larger GP1 subunit encompasses the glycan cap (GC), mucin-like domain (MLD) and receptor-binding site (RBS). It is believed that the heavily glycosylated GC and MLD participate in immune evasion by restricting the antibody access to GP1 core, including the RBS [8–10]. The GP2 subunit contains the hydrophobic internal fusion loop (IFL), two heptad repeats (HR1 and HR2), membrane-proximal external region (MPER) and transmembrane anchor [8]. After attachment to a cell membrane via low-affinity interactions, virions enter the cells by macropinocytosis mechanism [11]. At low pH inside endosomes, the cathepsins B and L cleave GP to remove GC and MLD, revealing RBS for the interaction with specific filovirus receptor, the Niemann-Pick C1 (NPC1) protein [12, 13]. This interaction triggers the fusion between viral and host membranes and release of the nucleocapsids into the cytoplasm.

GC-targeting mAbs alone are protective in animal challenge models and likely contribute to overall protection during natural infections [14–16] and for vaccine-mediated protection of challenged animals [17]. Although neutralizing activity is considered to be the major mechanism of protection by mAbs [18], there is increasing evidence that Fc effector function contributes to the control and clearance of filoviral infections [19–25], and even neutralizing mAbs may require Fc functions to confer optimal levels of protection [26]. Several therapeutic antibody combinations, including ZMapp [27], REGN-EB3 [28], FVM04/CA45 [29], MBP134^AF^ [30], rEBOV-520/548 [31], rEBOV-442/515 [32], and 1C3/1C11 [33], were generated and shown to be protective in the non-human primate challenge models. GC-specific antibodies are important components of the most of these combinations and are known to form a large portion of the humoral immune response to natural ebolavirus infection [34–36]. Since GC is dispensable for virus entry, antiviral mechanisms employed by mAbs targeting GC remain unclear, although a recent study suggests that GC-specific mAbs can indirectly inhibit GP proteolysis by shifting the MLD position and sterically occluding the cathepsin cleavage loop [37]. Other mechanisms independent of Fab-mediated virus neutralization have been proposed for GC-specific mAbs, including antibody-dependent cellular phagocytosis (ADCP) and activation of NK cells [18, 23].

Complement is a host defense system comprising more than 30 soluble protein factors and cell surface receptors in blood and other body fluids that interact to sense and respond to invading pathogens. This system can be activated through the classical, alternative or lectin pathway, but only the classical pathway is antigen- and antibody-dependent, thus bridging the innate and adaptive immune systems. The classical pathway is triggered by binding of C1q to the Fc domain of antigen-bound antibodies (typically, IgG1, IgG3 or IgM). The C1q molecule is an assembly of six heterotrimers, each with a binding site for Fc. In plasma, C1q forms a complex with C1r and C1s serine proteases. Once C1q has bound multiple Fc regions, C1r and C1s get activated resulting in cleavage of the complement proteins C4 and C2 into larger (C4b, C2a) and smaller (C4a, C2b) fragments. The larger fragments associate to form the C3 convertase, C4bC2a, which cleaves C3 into C3a and C3b. The latter binds covalently to reactive surfaces and “label” pathogens and infected cells for subsequent elimination via phagocytosis. C3b also interacts with C4bC2a complex forming the C5 convertase, C4bC2aC3b, which cleaves C5 into C5a and C5b. C5b is deposited onto the activating surface and initiates irreversible binding of C6, C7, C8 and multiple copies of C9 to form the membrane attack complex (MAC). MAC permeates the lipid bilayer, causing the lysis of antigen-expressing cells or enveloped viral particles [38–40].

Several studies suggested that the complement system is an important component of EBOV neutralization by antibodies during the natural infection [41] and immunization of experimental animals [42, 43]. Moreover, a recent Fc-engineering study illustrated the possibility of tuning the mAb protective potential *in vivo* by introduction of mutations regulating the activation of complement. Specifically, it was shown that KWES set of amino acid mutations in an Fc-fragment of VIC16 mAb, which upregulates complement activation, improves the protection of mice from EBOV infection compared to unmodified mAb [25]. However, the direct requirement of complement for mAb-mediated protection against filovirus infections has not been demonstrated.

In the present study, we compared complement activation by ebolavirus GP-specific mAbs with different epitope specificities. First, using an antibody-dependent complement deposition (ADCD) assay, GC-specific mAbs were shown to better induce C3 deposition compared to MPER mAbs. Second, we developed a complement-dependent cytotoxicity (CDC) assay and demonstrated that GC-specific mAbs stimulate killing of the target antigen-expressing cells by complement, an activity that can be inhibited by mAbs recognizing the other parts of GP, such as the MPER or base region. Using the chemical inhibitor of N-linked glycosylation, we further showed that N-linked glycans on the GP surface, while serving as part of the mAb epitope, can nevertheless downregulate mAb-mediated CDC activity. This finding represents a previously unknown mechanism of evasion of antiviral complement activity employed by EBOV. Finally, the depletion of complement in mice by injection of the cobra venom factor (CVF) impaired the survival of EBOV-challenged animals upon treatment with some mAbs specific to GC, but not to the MPER, indicating requirement of the functional complement system for effective protection by GC-specific mAbs. These results contribute understanding the mechanisms of virus-complement interplay and highlight an important role of the complement system in anti-EBOV activity of GP-specific mAbs. The obtained data can inform the selection of GC-specific mAbs for improved therapeutic antibody combinations.

## Results

### Glycan cap mAbs are more potent complement activators compared to MPER mAbs.

From our previously published studies on human mAbs isolated from survivors of ebolavirus infection [26, 34, 37, 44–46], we selected a panel of neutralizing GC- or MPER-specific mAbs to determine a possible difference in complement activation between these two groups of antibodies. A well-characterized 13C6 mouse mAb [42], which is a component of MB-003 and ZMapp combinations against EBOV and known to neutralize virus only in the presence of complement [20, 27], and mAbs ADI-15820 [35, 36] and KZ52 [47] isolated from human survivors of EBOV infection, also were included in this panel. For some antibodies, the recombinant versions bearing K322A (KA) mutation in the Fc fragment were produced, since this mutation greatly reduces binding to C1q resulting in the lack of efficient activation of complement [48].

**Figure 1:**
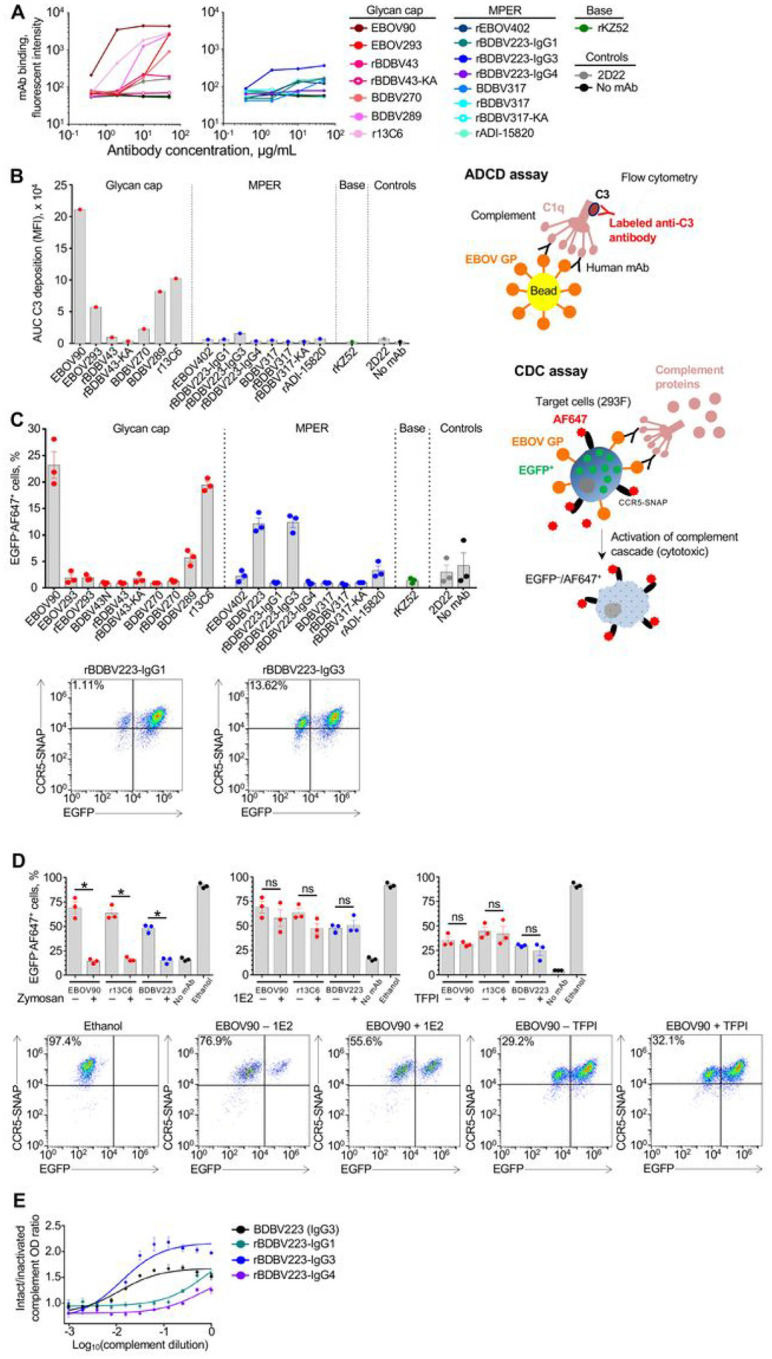
Glycan cap mAbs are more potent complement activators than MPER mAbs. Antibody-dependent complement deposition (ADCD) assay: (A) dose-response curves; (B) area under the curve values. GP-coated beads were incubated with mAbs and complement, and C3 deposition was quantified by flow cytometry. (C) Complement-dependent cytotoxicity (CDC) assay. 293F cells expressing EGFP, EBOV GP and the chimeric CCR5-SNAP tag protein were labeled with AF647 and incubated with mAbs (10 μg/mL) and complement (10%). The cytotoxicity was determined as percentage of EGFP–AF647+ cells by flow cytometry. (B) and (C) also include schematic representations of ADCD and CDC assays, respectively. Representative dot plots are shown. (D) Specificity of CDC assay. Complement was pre-treated with zymosan at 20 mg/mL (left) or 1E2 antibody at 0.1 mg/mL (middle) and added to cells, or 293F cells were treated with 1 μg/mL TFPI and incubated with complement (right). As controls, cells were mocktreated (no mAb), or treated with absolute ethanol (cell death control). *p 0.001; ns, not significant (unpaired t-test). Representative dot plots are shown. (E) Difference in complement activation by BDBV223 isotypes (10 μg/mL) validated by ELISA. Antigen-bound mAbs were incubated with serially diluted intact or heat-inactivated complement and the results were expressed as a ratio of C3-specific OD signals (OD intact compl. / OD heat-inact. compl). A, C-E: mean ± SEM of triplicate samples are shown.

First, we measured a dose-dependent mAb ability to induce C3 deposition onto GP-coated beads in ADCD assay (Fig. 1A, B). All the mAbs tested, with the exception of rBDBV223-IgG3 and -IgG4, belonged to the IgG1 subclass, allowing us to dissect the role of the epitope specificity in the activity. High levels of C3 deposition were observed for the GC-specific mAbs, whereas most of the tested mAbs specific to MPER, EBOV GP base region (KZ52), or irrelevant target (mAb (2D22; specific to dengue virus envelope protein [49]) did not show activity. We next asked whether the observed mAb effects on C3 deposition translated into the complement-mediated killing of antigen-expressing cells. For this work, we developed a CDC assay using the EBOV GPkik-293FS EGFP CCR5-SNAP cell line [50]. This cell line constitutively expresses EBOV GP on the plasma membrane, EGFP in the cytoplasm and the SNAP-tag CCR5, which can be specifically labeled with SNAP-Surface Alexa Fluor 647, on the cell surface. Fluorophore-labeled cells were incubated consecutively with the mAbs and complement, and the cytotoxicity was Quantified as a percentage of EGFP^−^AF647^+^ cells [51, 52] by analytical flow cytometry (Fig. 1C). Consistent with the data in Fig. 1A, B, CDC activity was observed for some of the GC-specific mAbs and with the hybridoma-produced and recombinant (rBDBV223-IgG3) versions of BDBV223 mAb, both belonging to the IgG3 subclass.

The specificity of the CDC assay was further validated in separate experiments using selected mAbs with high CDC activities. When complement was pre-treated with zymosan A, which was expected to consume the complement system activity [53], the cytotoxicity was significantly reduced for all antibodies regardless of the epitope specificity or IgG subclass (Fig. 1D, left). These results suggest that the cell killing activities observed for mAbs in the developed assay depends specifically on the presence of intact complement. Conversely, when complement was pre-treated with antibody 1E2 against the mannose-binding lectin (MBL), changes in mAb activity were not detected (Fig. 1D, middle). These data also were confirmed when tissue factor pathway inhibitor (TFPI) was added to cells along with the mAbs (Fig. 1D, right). TFPI is known as a selective inhibitor of MASP-2 serine protease of the lectin pathway, which does not affect the classical pathway proteases C1s or C1r [54]. Altogether, our data demonstrate that the observed mAb-driven cytotoxicity results from activation of the classical complement pathway.

IgG subclass-specific differences observed in ADCD and CDC assays were confirmed by an ELISA method for hybridoma-derived (IgG3) and recombinant (IgG1, IgG3, IgG4) versions of BDBV223 (Fig. 1E). In concordance with Fig. 1A–C data, the C3 deposition activity of BDBV223 mAb subclasses decreased in the following order: IgG3 > IgG1 > IgG4.

### MPER- and base-region-specific mAbs block cytotoxicity induced by GC or MPER mAbs.

**Figure 2:**
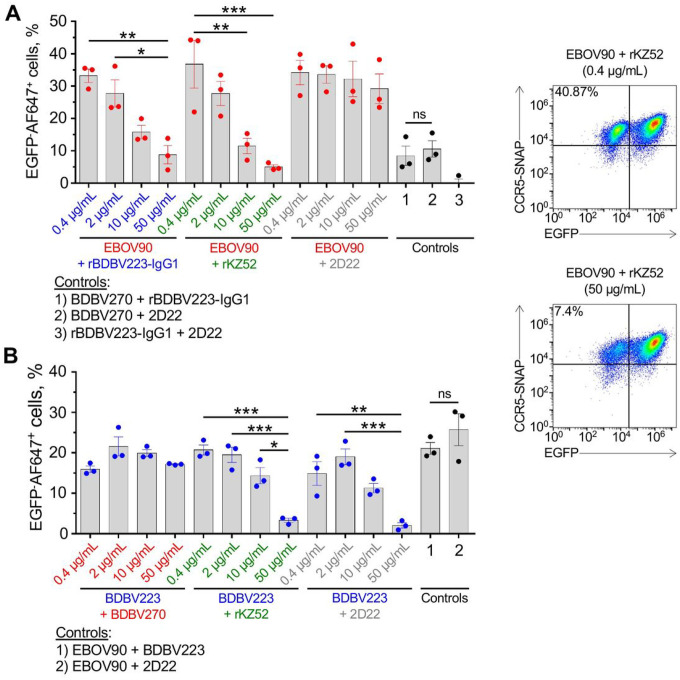
MPER and base mAbs block cytotoxicity induced by either GC or MPER mAbs. CDC assay with mixtures of (A) EBOV90 (10 μg/mL) or (B) BDBV223 mAb (10 μg/mL) with serially diluted mAbs of various epitope specificities at indicated concentrations (color-matched) was performed as in Fig. 1C. In control samples, mAbs were used at a concentration of 10 μg/mL each. Mean ± SEM of triplicate samples are shown. *p 0.05; **p 0.01; ***p 0.0001; ns, not significant (ANOVA, Tukey’s multiple comparisons test). Representative dot plots are shown.

We next tested if antibodies of various epitope specificities could interact to block or synergize each other’s CDC activities. In the first experiment, mixtures of GC-specific EBOV90 mAb with different concentrations of MPER (rBDBV223-IgG1), base (KZ52) or an irrelevant isotype-control antibody 2D22 were added to target cells (Fig. 2A). The CDC activity (the percentage of EGFP^−^AF647^+^ cells) of EBOV90 was dose-dependently inhibited by BDBV223 and KZ52, but not by 2D22, indicating that inactive MPER and base mAbs can reduce activities of GC-specific mAbs. At the same time, when BDBV223 was added to the GC mAb with low CDC activity (BDBV270), it did not change the CDC activity (Fig. 2A, controls).

In the second experiment, mixtures of the hybridoma-derived BDBV223 MPER mAb (IgG3 subclass) with different concentrations of a GC-specific (BDBV270), base-specific (KZ52) or irrelevant 2D22 mAb were tested (Fig. 2B). The CDC activity of BDBV223 was inhibited by KZ52, but not by BDBV270, in a dose-dependent manner. Surprisingly, the percentage of EGFP^−^AF647^+^ cells following incubation witth BDBV223 (IgG3) also was decreased when 50 μg/mL of 2D22 was added, suggesting some non-specific inhibition of the activity of BDBV223 by a maximal tested dose of an irrelevant antibody. When we mixed two highly active GC-specific mAbs of different epitope specificities, EBOV90 and MPER-specific BDBV223 (IgG3). no change in the CDC activity over the GC-specific antibody mixed with the 2D22 control mAb was observed (Fig. 2B, controls). The results of these experiments show that: 1) MPER-specific mAbs can inhibit CDC activity of the GC-specific mAbs, but not vice versa; 2) base-region-specific mAbs can inhibit the activity induced by both GC- and MPER-specific mAbs; and 3) mAbs targeting different EBOV GP epitopes do not show synergistic CDC activity.

### N-linked glycans on EBOV GP prevent CDC.

**Figure 3:**
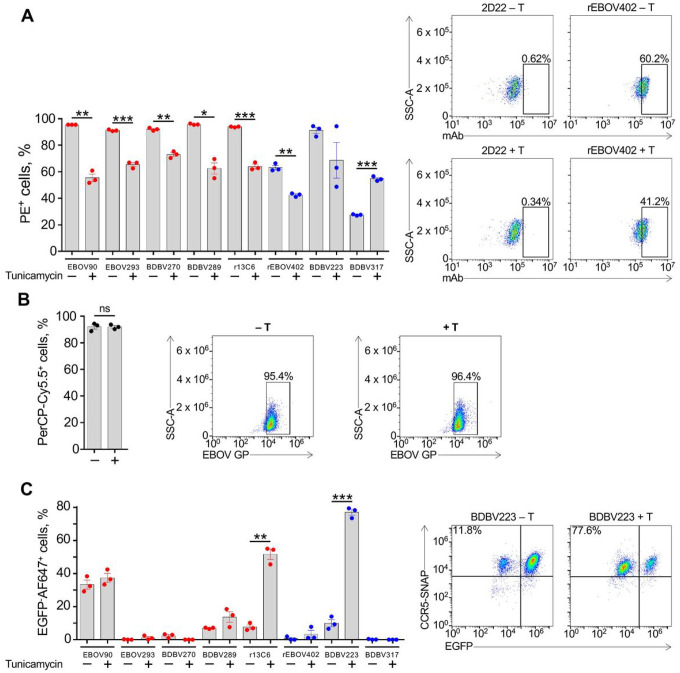
N-linked glycans on EBOV GP prevent activation of CDC mechanism. (A) SNAP-tagged 293F cells expressing EGFP and EBOV GP were treated with the vehicle control (−) or 1 μg/mL tunicamycin (+) and incubated with 10 μg/mL mAbs. Binding of mAbs to EBOV GP was determined by flow cytometry using PE-conjugated goat anti-human IgG secondary antibody. (B) SNAP-tagged 293F cells were treated as in (A), and the surface expression of EBOV GP was determined by flow cytometry using rabbit anti-EBOV VLP antiserum and mouse anti-rabbit IgG secondary antibody conjugated with PerCP-Cy5.5. (C) Cells were treated as in (A), and CDC assay was performed as in Fig. 1C. The percentages of EGFP–AF647+ cells in samples treated with the vehicle control or tunicamycin and incubated with 2D22 mAb were used for background signal subtraction. Mean ± SEM of triplicate samples are shown. *p 0.01; **p 0.001; ***p 0.0001; ns, not significant (unpaired t-test). Representative flow cytometry dot plots are shown.

EBOV GP is a heavily glycosylated protein [8, 55], which can affect multiple biological properties of the virus [56]. We next explored a possible role of N-glycans on EBOV GP in modulating mAb-induced CDC activity. First, we tested if pre-treatment of cells with tunicamycin, a chemical inhibitor of N-linked glycosylation [57], would alter mAb binding to EBOV GP. Tunicamycin treatment resulted in a significant decrease of mAb binding to 293F cells expressing EBOV GP, except for the BDBV317 MPER-specific mAb (Fig. 3A). At the same time, this treatment did not have a detectable effect on GP expression on the surface of target cells (Fig. 3B), suggesting that an impairment of mAb binding was not due to a reduction of GP expression level caused by tunicamycin. Interestingly, when the CDC assay was run for mAbs following tunicamycin treatment of cells, the opposite effect was observed: an increase, rather than decrease of activity, for some of the tested mAbs, regardless of the epitope specificity (Fig. 3C). Therefore, even though N-deglycosylation of GP disfavors mAb binding, it nevertheless results in hyperactivation of the complement-mediated lysis of target cells induced by mAbs. Overall, these data show that N-linked glycans on EBOV GP protect cells from CDC.

### GC-specific, but not MPER-specific mAbs, require complement for in vivo protection against EBOV.

**Figure 4:**
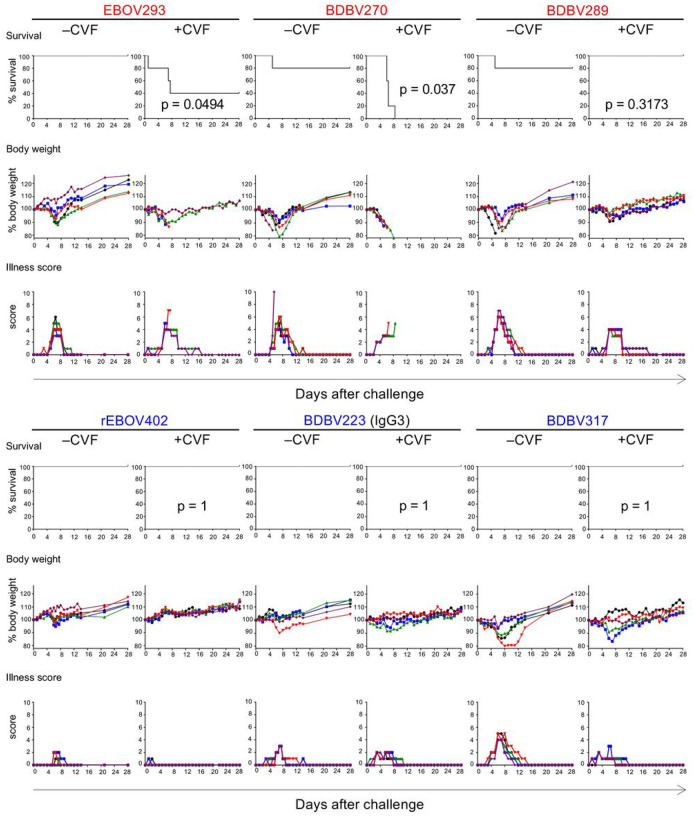
Glycan cap, but not MPER mAbs, require complement for in vivo protection against EBOV. Groups of mice at five animals per group were injected with indicated mAbs by the IP route at 24 h after EBOV challenge. Additionally, mice were treated with PBS (−CVF) or CVF (+CVF) one day prior to infection and at 3 dpi. Kaplan-Meier survival curves, body weight and illness score curves are shown. For each mAb, −CVF and +CVF groups are compared (Mantel-Cox test).

Finally, we addressed the role of the complement system in mAb-mediated protection against EBOV *in vivo*. Groups of BALB/c mice were treated with CVF to deplete their complement system, or mock-treated and next day exposed to a lethal (1,000 PFU) dose of mouse-adapted EBOV. On day 1 after infection mice were treated with individual mAbs at 100 μg (~5 mg/kg) or mock-treated, and on day 3 after infection, treatment or mock-treatment with CVF was repeated. For two out of three GC-specific mAbs tested, administration of CVF caused a significant impairment of protection (Fig. 4). Notably, for one of these mAbs, BDBV270, the protection was completely abrogated by CVF, as no animals in BDBV270/CVF group survived the infection. In contrast, full protection of mice against EBOV challenge was achieved by all MPER-specific mAbs tested, regardless of the CVF treatment. These data suggest that activation of the complement system is an important antiviral mechanism, which is required for *in vivo* protection conferred by mAbs targeting the GC but not the MPER of EBOV GP.

## Discussion

Using a combination of *in vitro* and *in vivo* approaches, we investigated the role of the complement system in antiviral mechanisms employed by antibodies directed to EBOV GP. First, using a bead-based ADCD assay, we compared the ability of two mAb groups with different epitope specificities to induce the C3 deposition on GP-coated surface. GC-specific mAbs were shown to be superior to their MPER-specific counterparts in ADCD activity (Fig. 1A, B). These data are in line with the results of a previous study, which analyzed multiple functional activities for 168 EBOV GP mAbs. Notably, antibodies targeting the most exposed GP regions, such as the head, GC and MLD, demonstrated stronger engagement of Fc-effector functions compared to mAbs against the conformationally obscured, “hidden” epitopes (*i.e*., HR2/MPER, IFL) [18, 23]. This observation was hypothesized to result from a greater accessibility of Fc fragments of mAbs bound to outer GP regions for the interaction with Fc receptors at the surface of immune cells, or with the complement system components [58].

Deposition of complement can lead to formation of MAC and lysis of lipid membranes of enveloped viruses [59] or infected host cells expressing viral antigens [60]. Deposition complement on virion particles may contribute to direct elimination of viral particles but probably is not critical for protection by mAbs that neutralize virus without complement [34, 37]. Elimination of infected cells by complement enhanced mechanisms, however, is more likely to reduce total viral burden. To test if the observed mAb ability to induce the C3 deposition on cells would result in an increased mAb-mediated cytotoxicity, we developed a CDC assay using a human-origin (human embryonic kidney 293F) cell line constitutively expressing EBOV GP [50] (Fig. 1C). The relative activity of individual mAbs in the CDC assay was similar to that determined by the ADCD assay, confirming the functional relevance of C3 deposition. High activity also was detected for an IgG3 form of the BDBV223 MPER mAb, the only antibody for which other subclasses in addition to IgG1 were tested. These results also were confirmed by ELISA (Fig. 1E). It is known that Fc-mediated activities vary greatly among IgG subclasses. The amino acid sequence of the C_H_2 region [61] and the antibody hinge region length [62] determines the complement-fixing potential of antibodies. IgG3 has the most potent affinity for binding to C1q, followed by IgG1, with a very weak association for IgG2 and no detectable interaction for IgG4 [40, 63]. From that perspective, BDBV223-IgG3 serves as a positive control in the tested panel.

The specificity of the 293F-cell-based CDC assay we developed was validated with complement-depleting or pathway-inhibiting compounds. First, using zymosan A, we showed that the mAb-mediated cytotoxicity requires the presence of intact complement. Zymosan is a carbohydrate substance extracted from yeast cell walls and is a potent activator of the alternative complement pathway. Zymosan can directly interact with properdin, the regulatory plasma glycoprotein produced by neutrophils which forms the stabilizing complex with C3bBb convertase (C3bBbP). After non-covalent attachment to the surface of zymosan particles, properdin binds C3b and initiates assembly of C3bBbP complexes, facilitating the prompt depletion of C3 complement component by the amplification convertase [64]. Second, using TFPI and anti-MBL antibody, we demonstrated that the observed CDC activity results from activation of the classical, but not lectin, complement pathway and, therefore, specifically requires the presence of GP-specific antibodies (Fig. 1D).

Next, considering the difference in complement activation by GC- and MPER-specific mAbs, we questioned the possible biological outcome of interaction between these two antibody groups, as should normally happen in a context of a polyclonal antibody response to EBOV infection and/or vaccination. We found that the KZ52 base-specific mAb dose-dependently inhibited CDC activity of the GC-specific mAb EBOV90 and the MPER mAb BDBV223 (IgG3) mAbs, and that BDBV223 (IgG1) mAb inhibited the activity of EBOV90 (Fig. 2). GC-specific antibodies enhanced binding of base mAbs to EBOV GP [31, 32, 37, 65]. Presumably, the EBOV90/KZ52 combination favors binding of the complement-inactive KZ52 mAb, therefore shifting the balance towards low/no complement activation. Regarding the BDBV223/KZ52 and EBOV90/BDBV223 pairs, it is possible that binding of one mAb can interfere with the binding of another one by a partial stochiometric hindrance of its epitope and/or by inducing conformational changes in the GP. Another possibility is that an inactive mAb interacts more strongly with GP, simply because of higher affinity for binding compared to that of the second mAb, thus creating unfavorable conditions for complement activation. The latter scenario can be especially true for the EBOV90/BDBV223 combination, considering the high reactivity of BDBV223 to EBOV GP (EC_50_ = 24 ng/mL; [34]). Importantly, analysis of the convalescent plasma IgG protein repertoire in a survivor of the 2013–2016 West African EBOV epidemic identified the GC, base region and head domain/RBS as the most abundantly recognized antigenic sites on GP [66]. A longitudinal study of B cell responses to natural EBOV infection revealed the persistence of IgG1, rapid decline of IgG3, late appearance of IgG4 and the absence of IgG2 antibodies specific to the viral GP [67]. Therefore, regulation of complement activation at polyclonal antibody level is likely to be a complex process, given the overall diversity of immunogenic epitopes and the dynamics of IgG subclass composition.

Filovirus GPs are heavily glycosylated with both N-linked and O-linked glycans, and glycans contribute from one-third to one-half of their molecular weight [68]. The glycosylation of ebolavirus GP is extremely heterogeneous, with some sites carrying over 40 unique glycan compositions, and it is even possible that the virion surface does not contain two copies of GP with the exact same glycosylation pattern [69]. There are up to 17 N-linked glycosylation sites in ebolavirus GP, 5 of which are located in GC [69]. GP1 N-glycans are suggested to participate in an immune evasion by shielding the epitopes from antibody recognition [55]. The GP2 subunit contains two N-linked glycosylation sites that contribute GP expression, stability, and cell entry [70, 71]. Given the important role of N-linked glycans in virus structure and life cycle, we addressed their possible effect on the mAb CDC activity using tunicamycin. Initially identified as a natural antibiotic, tunicamycin is now widely used for blocking N-linked glycosylation by inhibiting the transfer of UDP-N-acetylglucosamine to dolichol phosphate in the endoplasmic reticulum of eukaryotic cells [72–74]. For most of the tested mAbs, tunicamycin treatment reduced binding to GP (Fig. 3A). This finding was unexpected, since removal of glycans by mutagenesis was shown to enhance sensitivity of vesicular stomatitis virus (VSV) pseudotyped with EBOV GP (VSV/EBOV-GP) to neutralization by whole IgG purified from the serum of vaccinated or convalescent cynomolgus macaques [55, 70]. However, our data suggest that, for certain mAbs, N-linked glycans may be a part of their epitopes, rather than shielding the epitopes from immune recognition. In particular, the epitopes for mAbs 13C6 and BDBV289 contain N238 and N268 glycans, and the EBOV293 mAb epitope contains an N268 glycan [69]. Interestingly, removal of the N563 glycan site by mutagenesis enhanced VSV/EBOV-GP neutralization for some mAbs, while impairing it for the other mAbs [36]. In our panel, the only mAb that demonstrated an increased GP binding in the presence of tunicamycin was BDBV317. Its epitope can be shielded by a N618 glycan, which is located close to the escape mutation site identified for this mAb [44]. It should be noted that tunicamycin treatment does not allow dissection of the role of specific glycans, which can be studied in part by site-directed mutagenesis approaches [36, 55, 70, 71]. However, tunicamycin treatment has the advantage that it does not change the amino acid residue at the site of glycosylation, minimizing a possible impact on GP expression (Fig. 3B). Surprisingly, tunicamycin treatment not only did not reduce the CDC activity, as one could expect based on mAb binding data (Fig. 3A), but, instead, caused an increase of the cytotoxicity for some of the tested mAbs (Fig. 3C). To our knowledge, the phenomenon of specific downregulation of the classical complement pathway by N-linked glycans at the viral surface has not been described. A possibility is that EBOV employs GP glycosylation to reduce the antiviral complement activity.

Finally, we selected a few available GC- and MPER-specific mAbs, for which we have previously reported protection *in vivo* [26, 34, 37, 45], and addressed the role of the complement system using CVF treatment in the mouse model of EBOV infection. CVF shares structural and functional properties with C3. It also has C3b-like activity in forming the extremely stable CVF-dependent convertase, CVF, Bb, which cleaves C3 and C5 components [75]. Treatment of BALB/c mice with CVF depletes complement [76, 77]. We showed that CVF treatment significantly impaired the protection conferred by the GC-specific mAbs EBOV293 and BDBV270 but did not impair the protection by BDBV289 mAb or any MPER-specific mAb (Fig. 4). Interestingly, although EBOV293 and BDBV270 initiated C3 deposition (Fig. 1A, B), they did not demonstrate CDC activity (Fig. 1C). These data suggest that C3 opsonization mediated by these mAbs can potentially trigger complement-dependent antiviral mechanisms other than MAC assembly and lysis of antigen-expressing cells. Such alternative mechanisms can include phagocytosis of opsonized targets by complement receptor-bearing cells [39, 78]. BDBV289 is more potently neutralizing compared to mAbs BDBV270 and EBOV293 [34, 37], and was shown protect in mouse, guinea pig [34] and rhesus macaque [79] models of ebolavirus infection. It is therefore possible that BDBV289-mediated protection relies mainly on Fab-dependent virus neutralization and does not require complement activation. Similarly, MPER-specific antibodies can protect through direct virus neutralization, likely by interfering with the viral fusion machinery [80].

Ebolaviruses continue to pose a significant threat to public health by inducing outbreaks and epidemics of a highly lethal disease. Passive immunotherapy remains the most reliable therapeutic option for prophylaxis and post-exposure treatment. Therefore, understanding of protective mechanisms used by antibodies is critical to inform development of the most effective immunotherapeutic regimens and design of vaccines. In the present study, we addressed the antiviral mechanism for GC-binding mAbs. Using *in vitro* assays, we showed that 1) GC mAbs are superior to MPER mAbs in complement activation; 2) CDC activity can be dose-dependently inhibited by complement-inactive mAbs of different epitope specificity; 3) N-linked glycans can serve as a part of a mAb epitope and 4) N-linked glycans greatly downregulate CDC activity. Moreover, we were able to directly demonstrate the requirement of intact complement for *in vivo* protection conferred by GC-specific mAbs. Altogether, our results highlight the previously underappreciated role for activation of the complement system as an important mechanism of antibody-mediated protection against EBOV.

## Materials And Methods

### Cell lines.

293F cells expressing EBOV GP (strain Kikwit) on the plasma membrane, EGFP in the cytoplasm and the SNAP-tag CCR5 on the cell surface [50] were kindly provided by Dr. George K. Lewis (University of Maryland). The cell suspension was maintained in FreeStyle^™^ 293 expression medium (Gibco) containing 1 μg/mL puromycin (InvivoGen) at 37°C in 8% CO_2_ shaken at 130 rpm. Vero-E6 cells (green monkey kidney epithelial) were obtained from ATCC (CRL-1586). Cells were maintained in minimum essential medium supplemented with 10% fetal bovine serum and 1% penicillin-streptomycin solution (Gibco) at 37°C in 5% CO_2_.

### Viruses.

The mouse-adapted EBOV strain Mayinga (EBOV-MA, isolate EBOV/M.mus-tc/COD/76/Yambuku-Mayinga, GenBank accession number: AF499101) was originally generated by Dr. Mike Bray (U.S. Army Medical Research Institute of Infectious Diseases) [81]. The virus was provided originally by the Special Pathogens Branch of CDC, deposited in the World Reference Center for Emerging Viruses and Arboviruses at UTMB, and amplified by one passage in Vero-E6 cells. To determine the titer, virus was inoculated onto Vero-E6 cell culture monolayers, and incubated for 14 days under 0.45% methylcellulose (Thermo Fisher Scientific) overlay. Then, monolayers were fixed with formalin (Thermo Fisher Scientific), and viral plaques were immunostained with rabbit polyclonal antibody against EBOV GP (IBT Bioservices), Horse radish peroxidase (HRP)-labeled goat anti-rabbit IgG secondary antibody (Thermo Fisher Scientific) and Vector NovaRED peroxidase substrate kit (Vector Laboratories).

### Production of hybridoma-derived and recombinant mAbs.

Hybridoma mAbs EBOV90 (IgG1 isotype), BDBV270 (IgG1 isotype), EBOV293 (IgG1 isotype), BDBV317 (IgG1 isotype), BDBV223 (IgG3 isotype), and BDBV289 (IgG1 isotype) were isolated from a human survivor of a natural EBOV or BDBV infection as described previously [34, 37]. MAb 2D22 (IgG1 isotype) that is specific to dengue virus envelope (E) protein was described previously [82]. MAbs EBOV293, BDBV43, BDBV270, EBOV402, BDBV223, BDBV317 and mAbs ADI-15820 and KZ52 were produced in mammalian Expi293F or ExpiCHO cells (Gibco). ADI-15820 and KZ52 were produced based on known heavy- and light-chain variable region genes for these mAbs. Antibody heavy- and light-chain variable region genes were sequenced from hybridoma lines that had been cloned biologically by flow cytometric sorting. Briefly, total RNA was extracted using the RNeasy Mini kit (QIAGEN) and reverse-transcriptase PCR (RT-PCR) amplification of the antibody gene cDNAs was performed using the PrimeScript One Step RT-PCR kit (Takara Bio Inc.) according to the manufacturer’s protocol with gene-specific primers [83]. The thermal cycling conditions were as follows: 50°C for 30 min, 94°C for 2 min, 40 cycles of (94°C for 30 s, 58°C for 30 s and 72°C for 1 min). PCR products were purified using Agencourt AMPure XP magnetic beads (Beckman Coulter) and sequenced directly using an ABI3700 automated DNA analyzer. For recombinant mAb production, cDNA encoding the genes of heavy and light chains were cloned into DNA plasmid monocistronic expression vectors for mammalian cell culture mAb secretion encoding IgG1-, IgG3, IgG4, or IgG1-KA -heavy chain [84] and transformed into *Escherichia coli* cells. This vector contains an enhanced 2A sequence and GSG linker that enables simultaneous expression of mAb heavy- and light-chain genes from a single construct after transfection. MAb proteins were produced following transiently transfection of Expi293F or ExpiCHO cells following the manufacturer’s protocol and were purified from filtered culture supernatants by fast protein liquid chromatography on an ÄKTA instrument using HiTrap MabSelect Sure or HiTrap Protein G columns (GE Healthcare). Purified mAbs were buffer exchanged into phosphate buffered saline (PBS), filtered using sterile 0.45-μm pore size filter devices (Millipore), concentrated, and stored in aliquots at −80°C until use. Purification of hybridoma-produced mAbs is described elsewhere [85].

### Analysis of mAb IgG subclass specificity.

The isotype and subclass of secreted antibodies were confirmed by ELISA using murine anti-human IgG1, IgG3 or IgG4 mouse antibodies conjugated with alkaline phosphatase (Southern Biotech).

### Antibody mediated complement-(ADCD).

Recombinant EBOV GP with the transmembrane domain removed (GPΔTM) (Mayinga strain; IBT Bioservices) was biotinylated using LC-LC-Sulfo-NHS Biotin (Thermo Fisher Scientific). Excess biotin was removed using a Zeba desalting column (Thermo Fisher Scientific). Biotinylated GP antigen was then coupled to 1 μm red Neutravidin beads (Thermo Fisher Scientific) by incubating beads and antigen overnight at 4°C. Beads were washed twice with PBS containing 0.1% bovine serum albumin (BSA). mAbs were diluted in unsupplemented RPMI1640 (Gibco) and incubated with GP-coated beads for 2 h at 37°C. Unbound antibodies were removed by centrifugation prior to the addition of reconstituted guinea pig complement (Cedarlane Labs) diluted in veronal buffer supplemented with calcium and magnesium (Boston Bioproducts) for 20 min at 37°C. Beads were washed with PBS containing 15 mM EDTA and stained with an Fluorescein-5-isothiocyanate (FITC)-conjugated anti-guinea pig C3 antibody (MP Biomedicals). C3 deposition onto beads was measured using a BD LSRII flow cytometer (BD Biosciences). The geometric mean fluorescent intensity of FITC of all beads was measured. Data analysis was performed using FlowJo (BD Biosciences) Version X.

### C3c-specific ELISA.

Flat-bottom high-binding 96-well microplates (Greiner Bio-One) were coated at 4°C overnight with purified EBOV GP (Mayinga strain; Sino Biologicals) diluted at 1 μg/mL in Dulbecco’s phosphate-buffered saline (PBS; Corning) and washed four times with PBST buffer (0.1% Tween-20 in PBS). Bound antigen was blocked with 0.5% bovine serum albumin (BSA; Sigma-Aldrich) in PBST buffer for 30 min at room temperature. Then, blocking buffer was removed, and mAbs were added in triplicates at 10 μg/mL in PBST-0.5% BSA and the plates were incubated for 1 h at room temperature. Plates were washed four times in PBST, two-fold serial dilutions of human complement sera (Sigma-Aldrich) in PBST-0.5% BSA from 1:1 to 1:2,048 were added, and plates were incubated for 20 min at 37°C. Dilutions of heat-inactivated complement (30 min, 56°C) were added to control wells. After four washes with PBST, HRP-conjugated sheep anti-human C3c secondary antibody (Thermo Fisher Scientific) diluted at 1:500 in blocking buffer was added, and plates were incubated for 1 h at room temperature. Next, plates were washed four times in PBST, KPL SureBlue TMB peroxidase substrate solution (SeraCare) was added, and plates were incubated for 10 min at room temperature. The reaction was stopped by an equal volume of KPL TMB BLUESTOP solution (SeraCare), and plates were scanned in a Synergy microplate reader (BioTek) at the emission wavelength 630 nm. The results were expressed as a ratio of C3-specific OD signals after incubation of antigen-bound mAbs with serially diluted intact or heat-inactivated complement.

### Complement-dependent cytotoxicity (CDC) assay.

SNAP-tagged 293F cells expressing EGFP and EBOV GP (0.5 × 10^6^ cells per sample) were washed with PBS containing 1% BSA and incubated for 30 min with SNAP-Surface Alexa Fluor (AF) 647 substrate (New England BioLabs) at 37°C, 8% CO_2_, 130 rpm. Then, cells were washed three times with PBS and incubated in triplicates at room temperature with indicated concentrations of mAb or mAb mixtures diluted in FreeStyle^™^ 293 expression medium. In 15 min, baby rabbit complement (Cedarlane) was added up to a final concentration of 10%, and cells were incubated on a shaker for 6 h at 37°C, 8% CO_2_, 130 rpm, washed twice with PBS-1% BSA, fixed with 4% methanol-free formaldehyde solution (Thermo Fisher Scientific) and kept overnight at 4°C in dark. Next, cells were washed twice with PBS and analyzed by flow cytometry using an Accuri C6 cytometer (BD Biosciences). The cytotoxicity of the mAb was determined as the percentage of cells losing EGFP (by virtue of CDC) but retaining the surface expression of CCR5-SNAP (EGFP^−^AF647^+^).

In some experiments, the complement was pre-treated with 20 mg/mL zymosan A (Sigma-Aldrich) or 0.1 mg/mL 1E2 antibody (Abcam) for 1 h at 37°C before addition to cells, or TFPI (Sigma-Aldrich) was added to cells together with antibodies up to the final concentration of 1 μg/mL. Absolute ethanol was used as a cell death control.

In N-deglycosylation experiments, cells were treated overnight on a shaker at 37°C, 8% CO_2_, 130 rpm with 1 μg/mL tunicamycin (Sigma-Aldrich) diluted in ethanol, or treated with 0.1% ethanol (vehicle control), and then subjected to CDC assay. Same concentrations of tunicamycin or its diluent were maintained during incubation of cells with mAbs and complement. The percentages of EGFP^−^AF647^+^ cells in samples treated with the vehicle control or tunicamycin and incubated with 2D22 mAb were used for background signal subtraction.

### MAb binding to tunicamycin-treated 293F cells.

Cells were treated overnight with tunicamycin or vehicle control as described above, washed twice with PBS-1% BSA, and incubated in triplicates with 10 μg/mL mAbs diluted in PBS-1% BSA for 20 min at room temperature. Then, cells were washed twice with PBS-1% BSA and incubated with PE-conjugated goat anti-human IgG secondary antibody (Thermo Fisher Scientific) diluted at 1:200 in PBS-1% BSA for 20 min in dark at room temperature. After two washes with PBS-1% BSA, cells were fixed with 4% formaldehyde and kept overnight at 4°C in dark. Next, cells were washed twice with PBS, and the percentages of antibody-bound cells (PE^+^) were determined by flow cytometry as above. The percentages of PE^+^ cells in samples treated with the vehicle control or tunicamycin and incubated with 2D22 mAb were used for background signal subtraction.

### GP expression on tunicamycin-treated cells.

Cells were treated overnight with tunicamycin or vehicle control as described above, washed twice with PBS-1% BSA, and incubated in triplicates with rabbit anti-EBOV VLP antiserum (IBT Bioservices) diluted at 1:100 in PBS-1% BSA for 20 min at room temperature. Then, cells were washed twice with PBS-1% BSA and incubated with PerCP-Cy5.5-conjugated mouse anti-rabbit IgG secondary antibody (Santa Cruz Biotechnology) diluted at 1:200 in PBS-1% BSA for 20 min in dark at room temperature. After two washes with PBS-1% BSA, cells were fixed with 4% formaldehyde and kept overnight at 4°C in dark. Next, cells were washed twice with PBS, and percentages of GP expressing cells (PerCP-Cy5.5^+^) were determined by flow cytometry as above.

### Mouse studies.

Mice were housed in microisolator cages and provided food and water ad libitum. Groups of 7–8-week-old BALB/c mice (Charles River Laboratories) were inoculated with 1,000 PFU of the EBOV-MA by the intraperitoneal (i.p.) route in 100 μL PBS. Viral inoculate was back titrated at time of infection to verify viral titer. Mice (n = 5) were treated i.p. with 20 μg (or approximately 1 unit) of CVF (Sigma-Aldrich) in 500 μL PBS or mock-treated at one day prior to and three days after the challenge, and with 100 μg (~ 5 mg/kg) of individual mAb in 100 μL PBS on day 1 post-challenge. Mice were monitored twice daily from day 0 to day 14 post-challenge for illness, survival, and weight loss, followed by once daily monitoring from day 15 to the end of the study at day 28, as described elsewhere [86]. Moribund mice were euthanized as per the approved protocol (see Ethics statement). All mice were euthanized on day 28 after EBOV challenge.

### Statistical analysis.

Statistical analyses and generation of graphs were performed using GraphPad Prism version 6.07 (GraphPad Software). One-way ANOVA with multiple comparisons (Tukey’s test) or a T-test were used for statistical data analysis. Animal survival data were analyzed by log-rank (Mantel-Cox) test.

### Ethics statement.

Challenge studies were conducted under maximum containment in an animal biosafety level 4 facility of the Galveston National Laboratory, UTMB. The animal protocol was approved by the Institutional Animal Care and Use Committee in compliance with the Animal Welfare Act and other applicable federal statutes and regulations relating to animals and experiments involving animals.

## References

[1] Kuhn JH, Adkins S, Agwanda BR, Al Kubrusli R, Alkhovsky SV, Amarasinghe GK, 2021 Taxonomic update of phylum Negarnaviricota (Riboviria: Orthornavirae), including the large orders Bunyavirales and Mononegavirales. Archives of virology. 2021;166(12):3513–66. Epub 2021/09/01. doi: 10.1007/s00705-021-05143-6. 34463877 PMC8627462

[2] CDC. Cases and outbreaks of Ebola Virus Disease by Year. 2022. Available from: http://www.cdc.gov/vhf/ebola/outbreaks/history/chronology.html.

[3] CDC. 2014 Ebola Outbreak in West Africa - Case Counts. 2016. Available from: http://www.cdc.gov/vhf/ebola/outbreaks/2014-west-africa/case-counts.html.

[4] Mulangu S, Dodd LE, Davey RT Jr., Tshiani Mbaya O, Proschan M, Mukadi D, A Randomized, Controlled Trial of Ebola Virus Disease Therapeutics. The New England journal of medicine. 2019;381(24):2293–303. Epub 2019/11/28. doi: 10.1056/NEJMoa1910993. 31774950 PMC10680050

[5] FDA. FDA approves treatment for ebola virus 2020. Available from: https://www.fda.gov/drugs/news-events-human-drugs/fda-approves-treatment-ebola-virus.

[6] FDA. FDA Approves First Treatment for Ebola Virus 2020. Available from: https://www.fda.gov/news-events/press-announcements/fda-approves-first-treatment-ebola-virus.

[7] Volchkov VE, Feldmann H, Volchkova VA, Klenk HD. Processing of the Ebola virus glycoprotein by the proprotein convertase furin. Proc Natl Acad Sci U S A. 1998;95(10):5762–7. 9576958 10.1073/pnas.95.10.5762 PMC20453

[8] Lee JE, Fusco ML, Hessell AJ, Oswald WB, Burton DR, Saphire EO. Structure of the Ebola virus glycoprotein bound to an antibody from a human survivor. Nature. 2008;454(7201):177–82. 18615077 10.1038/nature07082 PMC2700032

[9] Lee JE, Saphire EO. Neutralizing ebolavirus: structural insights into the envelope glycoprotein and antibodies targeted against it. Curr Opin Struct Biol. 2009;19(4):408–17. doi: 10.1016/j.sbi.2009.05.004. 19559599 PMC2759674

[10] Lee JE, Saphire EO. Ebolavirus glycoprotein structure and mechanism of entry. Future Virol. 2009;4(6):621–35. doi: 10.2217/fvl.09.56. 20198110 PMC2829775

[11] Saeed MF, Kolokoltsov AA, Albrecht T, Davey RA. Cellular entry of ebola virus involves uptake by a macropinocytosis-like mechanism and subsequent trafficking through early and late endosomes. PLoS pathogens. 2010;6(9):e1001110. Epub 2010/09/24. doi: 10.1371/journal.ppat.1001110. 20862315 PMC2940741

[12] Chandran K, Sullivan NJ, Felbor U, Whelan SP, Cunningham JM. Endosomal proteolysis of the Ebola virus glycoprotein is necessary for infection. Science. 2005;308(5728):1643–5. 15831716 10.1126/science.1110656 PMC4797943

[13] Cote M, Misasi J, Ren T, Bruchez A, Lee K, Filone CM, Small molecule inhibitors reveal Niemann-Pick C1 is essential for Ebola virus infection. Nature. 2011;477(7364):344–8. Epub 2011/08/26. doi: 10.1038/nature10380. 21866101 PMC3230319

[14] Ksiazek TG, Rollin PE, Williams AJ, Bressler DS, Martin ML, Swanepoel R, Clinical virology of Ebola hemorrhagic fever (EHF): virus, virus antigen, and IgG and IgM antibody findings among EHF patients in Kikwit, Democratic Republic of the Congo, 1995. J Infect Dis. 1999;179 Suppl 1:S177–87. Epub 1999/02/13. doi: 10.1086/514321. 9988182

[15] Baize S, Leroy EM, Georges-Courbot MC, Capron M, Lansoud-Soukate J, Debre P, Defective humoral responses and extensive intravascular apoptosis are associated with fatal outcome in Ebola virus-infected patients. Nat Med. 1999;5(4):423–6. doi: 10.1038/7422. 10202932

[16] Reynard S, Journeaux A, Gloaguen E, Schaeffer J, Varet H, Pietrosemoli N, Immune parameters and outcomes during Ebola virus disease. JCI Insight. 2019;4(1). Epub 2019/01/11. doi: 10.1172/jci.insight.125106. PMC6485372 30626757

[17] Marzi A, Engelmann F, Feldmann F, Haberthur K, Shupert WL, Brining D, Antibodies are necessary for rVSV/ZEBOV-GP-mediated protection against lethal Ebola virus challenge in nonhuman primates. Proceedings of the National Academy of Sciences of the United States of America. 2013;110(5):1893–8. Epub 2013/01/16. doi: 10.1073/pnas.1209591110. 23319647 PMC3562844

[18] Saphire EO, Schendel SL, Fusco ML, Gangavarapu K, Gunn BM, Wec AZ, Systematic Analysis of Monoclonal Antibodies against Ebola Virus GP Defines Features that Contribute to Protection. Cell. 2018;174(4):938–52.e13. Epub 2018/08/11. doi: 10.1016/j.cell.2018.07.033. 30096313 PMC6102396

[19] Zeitlin L, Pettitt J, Scully C, Bohorova N, Kim D, Pauly M, Enhanced potency of a fucose-free monoclonal antibody being developed as an Ebola virus immunoprotectant. Proc Natl Acad Sci U S A. 2011;108(51):20690–4. doi: 10.1073/pnas.1108360108. 22143789 PMC3251097

[20] Olinger GG Jr., Pettitt J, Kim D, Working C, Bohorov O, Bratcher B, Delayed treatment of Ebola virus infection with plant-derived monoclonal antibodies provides protection in rhesus macaques. Proc Natl Acad Sci U S A. 2012;109(44):18030–5. doi: 10.1073/pnas.1213709109. 23071322 PMC3497800

[21] Holtsberg FW, Shulenin S, Vu H, Howell KA, Patel SJ, Gunn B, Pan-ebolavirus and Pan-filovirus Mouse Monoclonal Antibodies: Protection against Ebola and Sudan Viruses. Journal of virology. 2016;90(1):266–78. Epub 2015/10/16. doi: 10.1128/jvi.02171-15. 26468533 PMC4702560

[22] Misasi J, Gilman MS, Kanekiyo M, Gui M, Cagigi A, Mulangu S, Structural and molecular basis for Ebola virus neutralization by protective human antibodies. Science. 2016;351(6279):1343–6. doi: 10.1126/science.aad6117. 26917592 PMC5241105

[23] Gunn BM, Yu WH, Karim MM, Brannan JM, Herbert AS, Wec AZ, A Role for Fc Function in Therapeutic Monoclonal Antibody-Mediated Protection against Ebola Virus. Cell host & microbe. 2018;24(2):221–33.e5. Epub 2018/08/10. doi: 10.1016/j.chom.2018.07.009. 30092199 PMC6298217

[24] Ilinykh PA, Huang K, Santos RI, Gilchuk P, Gunn BM, Karim MM, Non-neutralizing Antibodies from a Marburg Infection Survivor Mediate Protection by Fc-Effector Functions and by Enhancing Efficacy of Other Antibodies. Cell host & microbe. 2020;27(6):976–91.e11. Epub 2020/04/23. doi: 10.1016/j.chom.2020.03.025. 32320678 PMC7292764

[25] Gunn BM, Lu R, Slein MD, Ilinykh PA, Huang K, Atyeo C, A Fc engineering approach to define functional humoral correlates of immunity against Ebola virus. Immunity. 2021;54(4):815–28.e5. Epub 2021/04/15. doi: 10.1016/j.immuni.2021.03.009. 33852832 PMC8111768

[26] Ilinykh PA, Santos RI, Gunn BM, Kuzmina NA, Shen X, Huang K, Asymmetric antiviral effects of ebolavirus antibodies targeting glycoprotein stem and glycan cap. PLoS Pathog. 2018;14(8):e1007204. doi: 10.1371/journal.ppat.1007204. following competing interest: PAI, AIF, JECJ and AB hold a patent, which covers the antibodies described in the manuscript. 30138408 PMC6107261

[27] Qiu X, Wong G, Audet J, Bello A, Fernando L, Alimonti JB, Reversion of advanced Ebola virus disease in nonhuman primates with ZMapp. Nature. 2014;514(7520):47–53. Epub 2014/08/30. doi: 10.1038/nature13777. 25171469 PMC4214273

[28] Pascal KE, Dudgeon D, Trefry JC, Anantpadma M, Sakurai Y, Murin CD, Development of Clinical-Stage Human Monoclonal Antibodies That Treat Advanced Ebola Virus Disease in Nonhuman Primates. J Infect Dis. 2018;218(suppl_5):S612–S26. Epub 2018/06/04. doi: 10.1093/infdis/jiy285. 29860496 PMC6249601

[29] Brannan JM, He S, Howell KA, Prugar LI, Zhu W, Vu H, Post-exposure immunotherapy for two ebolaviruses and Marburg virus in nonhuman primates. Nat Commun. 2019;10(1):105. Epub 2019/01/12. doi: 10.1038/s41467-018-08040-w. 30631063 PMC6328579

[30] Bornholdt ZA, Herbert AS, Mire CE, He S, Cross RW, Wec AZ, A Two-Antibody Pan-Ebolavirus Cocktail Confers Broad Therapeutic Protection in Ferrets and Nonhuman Primates. Cell host & microbe. 2019;25(1):49–58.e5. Epub 2019/01/11. doi: 10.1016/j.chom.2018.12.005. 30629918 PMC6341996

[31] Gilchuk P, Murin CD, Milligan JC, Cross RW, Mire CE, Ilinykh PA, Analysis of a Therapeutic Antibody Cocktail Reveals Determinants for Cooperative and Broad Ebolavirus Neutralization. Immunity. 2020;52(2):388–403.e12. Epub 2020/02/06. doi: 10.1016/j.immuni.2020.01.001. 32023489 PMC7111202

[32] Gilchuk P, Murin CD, Cross RW, Ilinykh PA, Huang K, Kuzmina N, Pan-ebolavirus protective therapy by two multifunctional human antibodies. Cell. 2021;184(22):5593–607.e18. Epub 2021/10/30. doi: 10.1016/j.cell.2021.09.035. 34715022 PMC8716180

[33] Milligan JC, Davis CW, Yu X, Ilinykh PA, Huang K, Halfmann PJ, Asymmetric and non-stoichiometric glycoprotein recognition by two distinct antibodies results in broad protection against ebolaviruses. Cell. 2022;185(6):995–1007.e18. Epub 2022/03/19. doi: 10.1016/j.cell.2022.02.023. 35303429 PMC10204903

[34] Flyak AI, Shen X, Murin CD, Turner HL, David JA, Fusco ML, Cross-Reactive and Potent Neutralizing Antibody Responses in Human Survivors of Natural Ebolavirus Infection. Cell. 2016;164(3):392–405. doi: 10.1016/j.cell.2015.12.022. 26806128 PMC4733404

[35] Bornholdt ZA, Turner HL, Murin CD, Li W, Sok D, Souders CA, Isolation of potent neutralizing antibodies from a survivor of the 2014 Ebola virus outbreak. Science. 2016;351(6277):1078–83. Epub 2016/02/26. doi: 10.1126/science.aad5788. 26912366 PMC4900763

[36] Wec AZ, Herbert AS, Murin CD, Nyakatura EK, Abelson DM, Fels JM, Antibodies from a Human Survivor Define Sites of Vulnerability for Broad Protection against Ebolaviruses. Cell. 2017;169(5):878–90.e15. Epub 2017/05/20. doi: 10.1016/j.cell.2017.04.037. 28525755 PMC5808922

[37] Murin CD, Gilchuk P, Ilinykh PA, Huang K, Kuzmina N, Shen X, Convergence of a common solution for broad ebolavirus neutralization by glycan cap-directed human antibodies. Cell Rep. 2021;35(2):108984. Epub 2021/04/15. doi: 10.1016/j.celrep.2021.108984. 33852862 PMC8133395

[38] Ricklin D, Hajishengallis G, Yang K, Lambris JD. Complement: a key system for immune surveillance and homeostasis. Nature immunology. 2010;11(9):785–97. Epub 2010/08/20. doi: 10.1038/ni.1923. 20720586 PMC2924908

[39] Murphy K, Weaver C. Janeway’s Immunobiology 9th ed. 2017.

[40] Mellors J, Tipton T, Longet S, Carroll M. Viral Evasion of the Complement System and Its Importance for Vaccines and Therapeutics. Frontiers in immunology. 2020;11:1450. Epub 2020/08/01. doi: 10.3389/fimmu.2020.01450. 32733480 PMC7363932

[41] Mellors J, Tipton T, Fehling SK, Akoi Bore J, Koundouno FR, Hall Y, Complement-Mediated Neutralisation Identified in Ebola Virus Disease Survivor Plasma: Implications for Protection and Pathogenesis. Front Immunol. 2022;13:857481. Epub 20220412. doi: 10.3389/fimmu.2022.857481. 35493467 PMC9039621

[42] Wilson JA, Hevey M, Bakken R, Guest S, Bray M, Schmaljohn AL, Epitopes involved in antibody-mediated protection from Ebola virus. Science. 2000;287(5458):1664–6. 10698744 10.1126/science.287.5458.1664

[43] Bukreyev AA, Dinapoli JM, Yang L, Murphy BR, Collins PL. Mucosal parainfluenza virus-vectored vaccine against Ebola virus replicates in the respiratory tract of vector-immune monkeys and is immunogenic. Virology. 2010;399(2):290–8. Epub 2010/02/05. doi: S0042-6822(10)00032-2 [pii] 10.1016/j.virol.2010.01.015. 20129638 PMC2842940

[44] Kuzmina NA, Younan P, Gilchuk P, Santos RI, Flyak AI, Ilinykh PA, Antibody-Dependent Enhancement of Ebola Virus Infection by Human Antibodies Isolated from Survivors. Cell reports. 2018;24(7):1802–15.e5. Epub 2018/08/16. doi: 10.1016/j.celrep.2018.07.035. 30110637 PMC6697154

[45] Flyak AI, Kuzmina N, Murin CD, Bryan C, Davidson E, Gilchuk P, Broadly neutralizing antibodies from human survivors target a conserved site in the Ebola virus glycoprotein HR2-MPER region. Nat Microbiol. 2018;3(6):670–7. doi: 10.1038/s41564-018-0157-z. 29736037 PMC6030461

[46] King LB, West BR, Moyer CL, Gilchuk P, Flyak A, Ilinykh PA, Cross-reactive neutralizing human survivor monoclonal antibody BDBV223 targets the ebolavirus stalk. Nat Commun. 2019;10(1):1788. Epub 2019/04/19. doi: 10.1038/s41467-019-09732-7. 30996276 PMC6470140

[47] Maruyama T, Rodriguez LL, Jahrling PB, Sanchez A, Khan AS, Nichol ST, Ebola virus can be effectively neutralized by antibody produced in natural human infection. J Virol. 1999;73(7):6024–30. Epub 1999/06/11. 10364354 10.1128/jvi.73.7.6024-6030.1999 PMC112663

[48] Idusogie EE, Presta LG, Gazzano-Santoro H, Totpal K, Wong PY, Ultsch M, Mapping of the C1q binding site on rituxan, a chimeric antibody with a human IgG1 Fc. Journal of immunology (Baltimore, Md: 1950). 2000;164(8):4178–84. Epub 2001/02/07. doi: 10.4049/jimmunol.164.8.4178. 10754313

[49] Fibriansah G, Ibarra KD, Ng TS, Smith SA, Tan JL, Lim XN, DENGUE VIRUS. Cryo-EM structure of an antibody that neutralizes dengue virus type 2 by locking E protein dimers. Science (New York, NY). 2015;349(6243):88–91. Epub 2015/07/04. doi: 10.1126/science.aaa8651. PMC4672004 26138979

[50] Domi A, Feldmann F, Basu R, McCurley N, Shiffl ett K, Emanuel J, A Single Dose of Modified Vaccinia Ankara expressing Ebola Virus Like Particles Protects Nonhuman Primates from Lethal Ebola Virus Challenge. Sci Rep. 2018;8(1):864. doi: 10.1038/s41598-017-19041-y. 29339750 PMC5770434

[51] Tolbert WD, Sherburn R, Gohain N, Ding S, Flinko R, Orlandi C, Defining rules governing recognition and Fc-mediated effector functions to the HIV-1 co-receptor binding site. BMC Biol. 2020;18(1):91. Epub 20200721. doi: 10.1186/s12915-020-00819-y. 32693837 PMC7374964

[52] Orlandi C, Flinko R, Lewis GK. A new cell line for high throughput HIV-specific antibody-dependent cellular cytotoxicity (ADCC) and cell-to-cell virus transmission studies. J Immunol Methods. 2016;433:51–8. Epub 20160308. doi: 10.1016/j.jim.2016.03.002. 26969387 PMC4869150

[53] Kouser L, Paudyal B, Kaur A, Stenbeck G, Jones LA, Abozaid SM, Human Properdin Opsonizes Nanoparticles and Triggers a Potent Pro-inflammatory Response by Macrophages without Involving Complement Activation. Front Immunol. 2018;9:131. Epub 20180212. doi: 10.3389/fimmu.2018.00131. 29483907 PMC5816341

[54] Keizer MP, Pouw RB, Kamp AM, Patiwael S, Marsman G, Hart MH, TFPI inhibits lectin pathway of complement activation by direct interaction with MASP-2. European journal of immunology. 2015;45(2):544–50. Epub 2014/11/02. doi: 10.1002/eji.201445070. 25359215

[55] Lennemann NJ, Rhein BA, Ndungo E, Chandran K, Qiu X, Maury W. Comprehensive functional analysis of N-linked glycans on Ebola virus GP1. MBio. 2014;5(1):e00862–13. doi: 10.1128/mBio.00862-13. 24473128 PMC3950510

[56] Vigerust DJ, Shepherd VL. Virus glycosylation: role in virulence and immune interactions. Trends Microbiol. 2007;15(5):211–8. doi: 10.1016/j.tim.2007.03.003. 17398101 PMC7127133

[57] Wu J, Chen S, Liu H, Zhang Z, Ni Z, Chen J, Tunicamycin specifically aggravates ER stress and overcomes chemoresistance in multidrug-resistant gastric cancer cells by inhibiting N-glycosylation. Journal of experimental & clinical cancer research: CR. 2018;37(1):272. Epub 2018/11/11. doi: 10.1186/s13046-018-0935-8. 30413206 PMC6230241

[58] Saphire EO, Schendel SL, Gunn BM, Milligan JC, Alter G. Antibody-mediated protection against Ebola virus. Nat Immunol. 2018;19(11):1169–78. Epub 2018/10/20. doi: 10.1038/s41590-018-0233-9. 30333617 PMC6814399

[59] Huber M, Fischer M, Misselwitz B, Manrique A, Kuster H, Niederöst B, Complement lysis activity in autologous plasma is associated with lower viral loads during the acute phase of HIV-1 infection. PLoS medicine. 2006;3(11):e441. Epub 2006/11/24. doi: 10.1371/journal.pmed.0030441. 17121450 PMC1637124

[60] Terajima M, Cruz J, Co MD, Lee JH, Kaur K, Wrammert J, Complement-dependent lysis of influenza a virus-infected cells by broadly cross-reactive human monoclonal antibodies. Journal of virology. 2011;85(24):13463–7. Epub 2011/10/14. doi: 10.1128/jvi.05193-11. 21994454 PMC3233150

[61] Tao MH, Canfield SM, Morrison SL. The differential ability of human IgG1 and IgG4 to activate complement is determined by the COOH-terminal sequence of the CH2 domain. J Exp Med. 1991;173(4):1025–8. doi: 10.1084/jem.173.4.1025. 2007852 PMC2190803

[62] Dangl JL, Wensel TG, Morrison SL, Stryer L, Herzenberg LA, Oi VT. Segmental flexibility and complement fixation of genetically engineered chimeric human, rabbit and mouse antibodies. EMBO J. 1988;7(7):1989–94. doi: 10.1002/j.1460-2075.1988.tb03037.x. 3138110 PMC454472

[63] Quast I, Peschke B, Lunemann JD. Regulation of antibody effector functions through IgG Fc N-glycosylation. Cell Mol Life Sci. 2017;74(5):837–47. doi: 10.1007/s00018-016-2366-z. 27639381 PMC11107549

[64] Spitzer D, Mitchell LM, Atkinson JP, Hourcade DE. Properdin can initiate complement activation by binding specific target surfaces and providing a platform for de novo convertase assembly. Journal of immunology (Baltimore, Md: 1950). 2007;179(4):2600–8. Epub 2007/08/07. doi: 10.4049/jimmunol.179.4.2600. 17675523

[65] Gilchuk P, Kuzmina N, Ilinykh PA, Huang K, Gunn BM, Bryan A, Multifunctional Pan-ebolavirus Antibody Recognizes a Site of Broad Vulnerability on the Ebolavirus Glycoprotein. Immunity. 2018;49(2):363–74.e10. Epub 2018/07/22. doi: 10.1016/j.immuni.2018.06.018. 30029854 PMC6104738

[66] Gilchuk P, Guthals A, Bonissone SR, Shaw JB, Ilinykh PA, Huang K, Proteo-Genomic Analysis Identifies Two Major Sites of Vulnerability on Ebolavirus Glycoprotein for Neutralizing Antibodies in Convalescent Human Plasma. Frontiers in immunology. 2021;12:706757. Epub 2021/08/03. doi: 10.3389/fimmu.2021.706757. 34335620 PMC8322977

[67] Davis CW, Jackson KJL, McElroy AK, Halfmann P, Huang J, Chennareddy C, Longitudinal Analysis of the Human B Cell Response to Ebola Virus Infection. Cell. 2019;177(6):1566–82.e17. Epub 2019/05/21. doi: 10.1016/j.cell.2019.04.036. 31104840 PMC6908968

[68] Sanchez A, Trappier SG, Mahy BW, Peters CJ, Nichol ST. The virion glycoproteins of Ebola viruses are encoded in two reading frames and are expressed through transcriptional editing. Proc Natl Acad Sci U S A. 1996;93(8):3602–7. 8622982 10.1073/pnas.93.8.3602 PMC39657

[69] Peng W, Rayaprolu V, Parvate AD, Pronker MF, Hui S, Parekh D, Glycan shield of the ebolavirus envelope glycoprotein GP. Communications biology. 2022;5(1):785. Epub 2022/08/05. doi: 10.1038/s42003-022-03767-1. 35927436 PMC9352669

[70] Lennemann NJ, Walkner M, Berkebile AR, Patel N, Maury W. The Role of Conserved N-Linked Glycans on Ebola Virus Glycoprotein 2. The Journal of infectious diseases. 2015;212 Suppl 2(Suppl 2):S204–9. Epub 2015/06/04. doi: 10.1093/infdis/jiv201. 26038399 PMC4564545

[71] Wang B, Wang Y, Frabutt DA, Zhang X, Yao X, Hu D, Mechanistic understanding of N-glycosylation in Ebola virus glycoprotein maturation and function. The Journal of biological chemistry. 2017;292(14):5860–70. Epub 2017/02/16. doi: 10.1074/jbc.M116.768168. 28196864 PMC5392578

[72] Surani MA. Glycoprotein synthesis and inhibition of glycosylation by tunicamycin in preimplantation mouse embryos: compaction and trophoblast adhesion. Cell. 1979;18(1):217–27. Epub 1979/09/01. doi: 10.1016/0092-8674(79)90370-2. 509524

[73] Hakulinen JK, Hering J, Brändén G, Chen H, Snijder A, Ek M, MraY-antibiotic complex reveals details of tunicamycin mode of action. Nature chemical biology. 2017;13(3):265–7. Epub 2017/01/10. doi: 10.1038/nchembio.2270. 28068312

[74] Yoo J, Mashalidis EH, Kuk ACY, Yamamoto K, Kaeser B, Ichikawa S, GlcNAc-1-P-transferase-tunicamycin complex structure reveals basis for inhibition of N-glycosylation. Nature structural & molecular biology. 2018;25(3):217–24. Epub 2018/02/21. doi: 10.1038/s41594-018-0031-y. PMC5840018 29459785

[75] Vogel CW, Fritzinger DC. Cobra venom factor: Structure, function, and humanization for therapeutic complement depletion. Toxicon: official journal of the International Society on Toxinology. 2010;56(7):1198–222. Epub 2010/04/27. doi: 10.1016/j.toxicon.2010.04.007. 20417224

[76] Van den Berg CW, Aerts PC, Van Dijk H. In vivo anti-complementary activities of the cobra venom factors from Naja naja and Naja haje. J Immunol Methods. 1991;136(2):287–94. 1999656 10.1016/0022-1759(91)90015-8

[77] Bukreyev A, Yang L, Collins PL. The secreted G protein of human respiratory syncytial virus antagonizes antibody-mediated restriction of replication involving macrophages and complement. Journal of virology. 2012;86(19):10880–4. Epub 2012/07/28. doi: 10.1128/jvi.01162-12. 22837211 PMC3457292

[78] Brown EJ. Complement receptors and phagocytosis. Current opinion in immunology. 1991;3(1):76–82. Epub 1991/02/01. doi: 10.1016/0952-7915(91)90081-b. 1675856

[79] Gilchuk P, Mire CE, Geisbert JB, Agans KN, Deer DJ, Cross RW, Efficacy of Human Monoclonal Antibody Monotherapy Against Bundibugyo Virus Infection in Nonhuman Primates. J Infect Dis. 2018;218(suppl_5):S565–S73. Epub 2018/07/10. doi: 10.1093/infdis/jiy295. 29982718 PMC6249568

[80] Hargreaves A, Brady C, Mellors J, Tipton T, Carroll MW, Longet S. Filovirus Neutralising Antibodies: Mechanisms of Action and Therapeutic Application. Pathogens (Basel, Switzerland). 2021;10(9). Epub 2021/09/29. doi: 10.3390/pathogens10091201. PMC8468515 34578233

[81] Bray M, Davis K, Geisbert T, Schmaljohn C, Huggins J. A mouse model for evaluation of prophylaxis and therapy of Ebola hemorrhagic fever. J Infect Dis. 1998;178(3):651–61. Epub 1998/09/05. 9728532 10.1086/515386

[82] Fibriansah G, Ibarra KD, Ng TS, Smith SA, Tan JL, Lim XN, Cryo-EM structure of an antibody that neutralizes dengue virus type 2 by locking E protein dimers. Science. 2015;349(6243):88–91. doi: 10.1126/science.aaa8651. 26138979 PMC4672004

[83] Thornburg NJ, Zhang H, Bangaru S, Sapparapu G, Kose N, Lampley RM, H7N9 influenza virus neutralizing antibodies that possess few somatic mutations. J Clin Invest. 2016;126(4):1482–94. doi: 10.1172/JCI85317. 26950424 PMC4811156

[84] Gilchuk P, Bombardi RG, Erasmus JH, Tan Q, Nargi R, Soto C, Integrated pipeline for the accelerated discovery of antiviral antibody therapeutics. Nat Biomed Eng. 2020;4(11):1030–43. Epub 2020/08/05. doi: 10.1038/s41551-020-0594-x. 32747832 PMC7655621

[85] Flyak AI, Ilinykh PA, Murin CD, Garron T, Shen X, Fusco ML, Mechanism of human antibody-mediated neutralization of Marburg virus. Cell. 2015;160(5):893–903. Epub 2015/02/28. doi: 10.1016/j.cell.2015.01.031. 25723164 PMC4344968

[86] Ilinykh PA, Graber J, Kuzmina NA, Huang K, Ksiazek TG, Crowe JE Jr., Ebolavirus Chimerization for the Development of a Mouse Model for Screening of Bundibugyo-Specific Antibodies. J Infect Dis. 2018;218(suppl_5):S418–S22. Epub 2018/07/31. doi: 10.1093/infdis/jiy423. 30060231 PMC6249583

